# The EM Method in a Probabilistic Wavelet-Based MRI Denoising

**DOI:** 10.1155/2015/182659

**Published:** 2015-05-18

**Authors:** Marcos Martin-Fernandez, Sergio Villullas

**Affiliations:** ^1^Laboratorio de Procesado de Imagen, Escuela Técnica Superior de Ingenieros de Telecomunicación, Campus Miguel Delibes s.n., 47011 Valladolid, Spain; ^2^Departamento de Álgebra, Análisis Matemático, Geometría y Topología, Facultad de Ciencias, Campus Miguel Delibes s.n., 47011 Valladolid, Spain

## Abstract

Human body heat emission and others external causes can interfere in magnetic resonance image acquisition and produce noise. In this kind of images, the noise, when no signal is present, is Rayleigh distributed and its wavelet coefficients can be approximately modeled by a Gaussian distribution. Noiseless magnetic resonance images can be modeled by a Laplacian distribution in the wavelet domain. This paper proposes a new magnetic resonance image denoising method to solve this fact. This method performs shrinkage of wavelet coefficients based on the conditioned probability of being noise or detail. The parameters involved in this filtering approach are calculated by means of the expectation maximization (EM) method, which avoids the need to use an estimator of noise variance. The efficiency of the proposed filter is studied and compared with other important filtering techniques, such as Nowak's, Donoho-Johnstone's, Awate-Whitaker's, and nonlocal means filters, in different 2D and 3D images.

## 1. Introduction

Magnetic resonance imaging (MRI) is one of the most important imaging acquisition techniques [1], which allows studying the structural features of the internal body parts noninvasively. This procedure is based on the principle of nuclear magnetic resonance (NMR) [2], and the power of this technique over other noninvasive techniques, such as ultrasound, is the high quality of its images, despite the inconvenience of being a larger and expensive equipment. However, because the ultrasonic signal is not transmitted through bones, in cases such as imaging the brain which is surrounded by the skull, ultrasound modalities are not viable and resorting to magnetic resonance (MR) imaging is needed.

For a given acquisition time, in MRI there is a fundamental agreement between resolution and signal to noise ratio (SNR) [3]. MRI is affected by noise mainly produced by interference due to human body heat emission (Gaussian at frequency space and Rayleigh at envelope of its inverse Fourier transform [4]), which prevents correct identification of shapes and details. Moreover, there is a relationship between noise level and image resolution in MRI acquisition, that is, the larger the resolution of the acquired image, the lower the SNR [5]. The simplest method to reduce the noise level is to increase the acquisition time of the machine (thus increasing the number of images averaged, that is, increasing the machine number of experiments (NEX)), which would cause a large increase in spending and long waiting lists, but long acquisition times could be problematic for patients who are not able to remain in a resting state (due to stress, pain, and so on). To avoid these problems, a filter, which acts on the acquired image, can be applied. This filter must eliminate noise trying to preserve details. The main problem of removing noise by means of softening an image is the resulting loss of information on the edges and contours (image blur), which is typical in Gaussian filter convolution. Perona and Malik [6] proposed a new type of filtering, based on partial differential equations and the heat diffusion equation, which is the origin of a family of filters that allow homogenizing regions while maintaining or enhancing borders between them. Gerig et al. [7] propose the use of the so-called nonlinear anisotropic filter, which gives very good results in the context of MRI and is an important practical application of the ideas proposed by Perona and Malik. From the same diffusion equation, [7] proposes an alternative discretization with simpler formulation, whose stability is subject to certain restrictions of the parameters. Linearly optimal methods in the sense of minimum mean square error (Wiener filter) have also been adapted to the case of MRI [8, 9]. Another filter with good MR image denoising results is the so-called nonlocal means (NLM) filter [10, 11], which averages similar image pixels as a function of their intensity distance (some filters, like the bilateral filter [4], are based on the same proposition, but the advantage of the NLM over other methods is that the similarity measure used is more robust to noise due to region comparisons rather than pixel comparisons). Principles of nonparametric statistical methods are also the base of the iterated conditional entropy reduction (ICER) proposed by Awate and Whitaker [12], a Bayesian-inference algorithm based on Markov random field which estimates the uncorrupted-image statistics by optimizing an information-theoretic metric using the expectation-maximization algorithm. This filter method incorporates a Rician noise model, unlike NLM method, which is more general. He and Greenshields [13] designed another filter method that improves NLM filter by adding Rician noise information. Dabov et al. [14] also proposed a filter similar to NLM. This method creates 3D arrays formed by stacking together similar image 2D neighborhoods. The importance of grouping is to enable the use of a higher-dimensional filtering of each group, which exploits the potential similarity (correlation, affinity, etc.) between grouped fragments. More generally, Sivaramakrishnan and Weissman [15] designed a universal filter that does not need* a priori* noise information which is asymptotically optimal. Furthermore, Awate and Whitaker also proposed a patch-based method [16, 17] that tries to optimize the entropy of the noisy image to reduce noise.

A large interesting characteristic of the wavelet transform is its capacity to preserve detail at different scales, because of its ability to model the information locally present in the image due to the multiresolution decomposition [18]. In the literature, other authors have performed MR image filtering using wavelet techniques. Donoho and Johnstone [19] proved that a simple thresholding algorithm with an appropriated base may be a (nonlinear) filter which is almost optimal. Nowak [20] and Pizurica et al. [21] propose to perform the filtering using the discrete wavelet transform. In particular, the significance of Nowak's work is that it uses the fact that MR magnitude image obeys a Rician distribution and its square image noise obeys a noncentral Chi-square distribution. In addition, other researches, like Sijbers et al. [22], use this fact. A diffusion method such as Perona-Malik's, but adapted to the Rician distribution case, has been proposed in [23]. In [20], the wavelet transform is performed on the square of the amplitude image where the noise and the bias of approximation coefficients are reduced. Another interesting work is that of Anand and Sahambi [4]. In this case, the square of the amplitude image is also used, correcting the bias and applying a bilateral filter (Gaussian filtering in the spatial and amplitude domains) over approximation coefficients (it is also based on the Rician image distribution). On the other hand, Yang and Fei [24] combined the 1D wavelet transform with the Radon transform to denoise Rician noise in MR images. Finally, we can also mention the method proposed by Wirestam et al. [25], where a technique of shrinkage coefficients of the filter is based on a Wiener filter. The novelty of this method is that the filtering is performed in the complex Fourier image, where noise data are complex Gaussian. This method has the problem that complex data are not always available in MRI acquisition (usually, MR machines provide only data in the image plane after envelope calculation in DICOM format. Complex raw data in the *k*-space are normally stored in a proprietary format which is not open and is brand-dependent. The complex data in the image plane are not even stored in the machine).

Based on wavelets state of art and given locality property of wavelet transform, the alternative that we propose in this paper is to perform filtering in the domain of the transform coefficients, adapting to MRI the method proposed in [26], originally designed for mammographic images. We also propose to estimate the parameters of the model by means of the expectation maximization (EM) method, proposed by Dempster et al. [27] and Moon [28]. Moreover, this fact makes the filter independent of noise variance estimators, in contrast to other filter methods.

This paper is structured as follows. In Section 2, some different MR image denoising methods are detailed. First, in Section 2.1, the methods proposed by Donoho and Johnstone [19] (Section 2.1.1) and Nowak [20] (Section 2.1.2) are described.  Section 2.1.3 shows shift variance of wavelets coefficients and how to correct it. This section also contains Section 2.2 (with Sections 2.2.1 and 2.2.2), where Awate-Whitaker's algorithm [16, 17] and nonlocal means filter [10, 11] are presented. Finally, Section 2.3 describes the used technique to estimate noise variance. In Section 3, the new wavelet denoising method, based on [26], is presented.  Section 4 presents the algorithms corresponding to the methods showed in Sections 2 and 3 and some practical experiments. First, Section 4.1 shows a step-by-step explanation of the different denoising algorithms presented before. Second, in Section 4.2, the images used to test the efficiency of the proposed method are detailed. The measurements used to compare the different filter methods are described in Section 4.3. Fourth, in Section 4.4, the numerical experiments and some remarks, obtained after performing these experiments, are presented. Finally, Section 5 contains a brief summary of the obtained results and some possible future research lines.  Appendix A contains the details about the optimization method proposed to estimate the parameters of the filter presented in Section 3 and Appendix B contains some useful auxiliary functions.

## 2. State of Art

This section reviews some filters used in the practical experiments of Section 4. The new filter proposed in this paper is compared with other two wavelet filters, Donoho-Johnstone's hard thresholding filter [19] and Nowak's [20] filter, the Awate and Whitaker's [16, 17] patch-based (Gaussian and Rician) filters and the nonlocal means [10, 11] filter. Moreover, wavelet-based filtering is shift-variant, as Section 2.1.3 shows. This subsection also presents how to avoid this problem. Finally, an estimator of noise variance, required in both wavelet-based filters, is given in Section 2.3.

### 2.1. Wavelet-Domain Filters

An image/volume can be interpreted as a 2-dimensional/3-dimensional function with compact support. The values of this function, represented in a matrix/3D array *I*, are a good approximation to scale coefficients, *s*_0_, in discrete wavelet transform. The fast wavelet transform algorithm let us calculate the scale, *s*_*j*_, and detail, *d*_*j*_, coefficients in the following levels 0 ≤ *j* ≤ *J*, that is, if *s*_0_ = *I*, *s*_*j*+1_ and *d*_*j*+1_ can be calculated in function of *s*_*j*_. Noise interferences modify the details of the MR image/volume; as noise grows more levels are affected. The wavelet coefficients are filtered to denoise the image/volume. The wavelet coefficients  *d* ^*α*^[*k*] (the index *α* corresponds to level and orientation: horizontal, vertical, diagonal, and so on, and index *k* corresponds to scale and position) can be determined by means of

(1) $$\begin{array}{c} \;d^{\alpha }\left[k\right]∶=\overset{}{\underset{m}{\sum }}\psi^{\alpha }\left[k\right]\left(m\right)I\left(m\right), \end{array}$$

where *ψ* ^*α*^[*k*] is the discrete wavelet function at *α* level and orientation and *k* scale and position, and index *m* represents pixel/voxel position. Similarly the scaling coefficient *s* ^*j*^[*k*] (the index *j* corresponds to level and index *k* corresponds to scale and position) can be determined by means of

(2) $$\begin{array}{c} s^{j}\left[k\right]∶=\underset{m}{\sum }\varphi^{j}\left[k\right]\left(m\right)I\left(m\right), \end{array}$$

where *φ* ^*j*^[*k*] is the scaling function for level *j* and *k* scale and position. Given a sequence of wavelet coefficients *d* ^*α*^ = (*d* ^*α*^[*k*])_*k*=1_^*N*^*α*^^ and scaling coefficients *s* ^*j*^ = (*s* ^*j*^[*k*])_*k*=1_^*N*^*j*^^ for the image/volume *I* (where *N* ^*α*^ represent the number of scales and positions for each *α* and *N* ^*j*^ is the number scales and positions for each *j*) the filters can be defined in the wavelet domain.

The following two filters, proposed by Donoho and Johnstone and Nowak, are used to analyze the efficiency of the new filter presented in next section.

#### 2.1.1. Donoho-Johnstone's Filter

The classic hard thresholding filter described by Donoho and Johnstone [19] is given by

(3) $$\begin{array}{c} F_{\text{DJ}}\left(d^{\alpha }\left[k\right]\right)∶=\left\{\begin{array}{cc} d^{\alpha }\left[k\right], & \text{if}\;\left|d^{\alpha }\left[k\right]\right|>T^{\alpha },\\ 0, & \text{if}\;\left|d^{\alpha }\left[k\right]\right|\leq T^{\alpha }, \end{array}\right. \end{array}$$

where |·| is the module operator and $T^{\alpha }∶=\sigma_{\text{noise}}\sqrt{2\text{lo}{\text{g}}_{e}(N^{\alpha })}$ with *σ*_noise_ standard deviation of the noise in the image/volume *I*.

#### 2.1.2. Nowak's Filter

Another filter is proposed by Nowak [20] by

(4) $$\begin{array}{c} F_{N}\left(d^{\alpha }\left[k\right]\right)∶=C^{\alpha }\left[k\right]d^{\alpha }\left[k\right], \end{array}$$

where

(5) Cαk∶=dαk2−3σα2kdαk2+,   x+∶=x,if  x≥0,0,if  x<0,

and (*σ* ^2^)^*α*^[*k*] is the variance of the wavelet coefficient *d* ^*α*^[*k*]. An estimation for that, $\hat{{\left(\sigma^{\alpha }\right)}^{2}[k]}$, is proposed in [20]

(6) $$\begin{array}{c} \hat{{\left(\sigma^{\alpha }\right)}^{2}\left[k\right]}∶=4\sigma_{\text{noise}}^{4}\max\left[\frac{\sum_{m\in I}^{}{\left(\psi^{\alpha }\right)}^{2}\left[k\right]\left(m\right)I^{2}\left(m\right)}{\sigma_{\text{noise}}^{2}}-1,1\right], \end{array}$$

where *σ*_noise_ is the standard deviation of the noise in the image/volume *I*.

#### 2.1.3. Shift-Invariant Filtering

**Figure 1:**
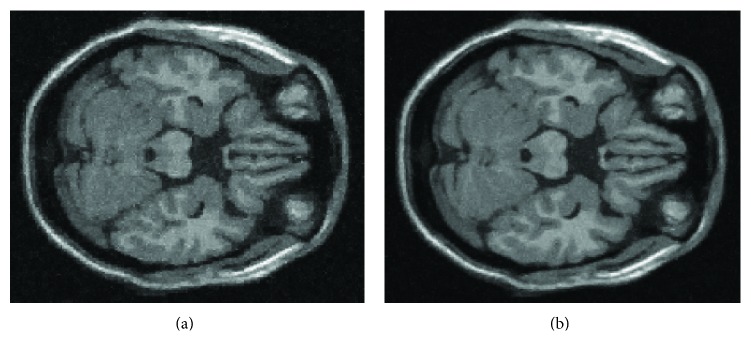
Comparative images: (a) without shift-invariant filtering; (b) with shift-invariant filtering (*K* = 2). Saw tooth (artifacts) can be seen in the case (a).

As [20] shows, wavelet coefficients filtering (based on discrete wavelet transform) is shift-variant, which can produce the appearance of artifacts, because the wavelet coefficient values depend on the alignment between the data and the wavelet basis functions. Shift-invariant (translation-invariant, undecimated) methods [29–33] can provide better performance, avoiding the appearance of artifacts (see an example in Figure 1). Shift-invariant wavelet transforms provide a higher degree of regularity [29, 33, 34] than standard wavelet analysis approaches, so shift-invariant estimation algorithms usually outperform standard methods. A shift-invariant filtering can be obtained by applying the filter for every possible shift of the image, unshifting each filtered result, and averaging all the results obtained. As performing all shifts of the image would be computationally too expensive, to reduce computational burden of filtering, an approximately shift-invariant scheme is proposed; that is, all shifts are replaced by a small range of shifts. More specifically, the image is shifted in both horizontal and vertical directions (left, right, up, and down), at most *K* pixels/voxels in each direction in steps of one pixel/voxel. While this does not guarantee shift-invariance, it does reduce the dependence of the filter output on the alignment between the data and the wavelet basis functions. In our experiments, we used *K* = 2, that is, movements of {−2, −1,0, 1,2} in each direction giving rise to a total of 25 shiftings for the 2D images (the values *K* > 2 enlarge computational time excessively with minor improvements), and *K* = 1, that is, movements of {−1,0, 1} in each direction giving rise to a total of 27 shiftings for the 3D volumes (the same remark as in 2D also applies in 3D for *K* > 1). The final image/volume is constructed after unshifting each image/volume and averaging the 25/27 resulting images/volumes.

### 2.2. Awate-Whitaker's Patch-Based Filter and Nonlocal Means Filter

For quantitative evaluation, these two wavelet methods (and the new proposed filter) are compared (in 2D case) with a patch-based method proposed by Awate and Whitaker [16, 17] and the nonlocal means (NLM) filter [10, 11]. This comparison will be very significative as this method was recently shown to be competitive with respect to wavelet methods.

#### 2.2.1. Awate-Whitaker's Filter

In this approach, given an image *I*, a random vector *Z*(*m*) = [*X*(*m*), *Y*(*m*)], where *m* represents the pixel position on the image *I*, is generated, where *X*(*m*) is the intensity of *I* at the pixel *m* and *Y*(*m*) is the intensity of *I* at the pixels in a neighborhood (*Y*(*m*) is a vector) of pixel *m* (we use $\tilde{X}(m)$,  $\tilde{Y}(m)$, and $\tilde{Z}(m)$ for degraded image random variables). The target of the method is to minimize the entropy of the conditional PDF, $h(\tilde{X}|\tilde{Y})$, for which a descending gradient method, given by

(7) $$\begin{array}{c} \hat{x}=x-\delta \frac{\partial h}{\partial x}, \end{array}$$

where *δ* is a parameter, is used. In this paper, we used two versions of this filter method using Gaussian and Rician models for the PDF.

#### 2.2.2. Nonlocal Means Filter

For a given image *I*, the NLM filtered image at pixel position *m* (*F*_NLM_ is the NLM filter operator) is given by the weighted average of all the pixels in a searched area *Ω*_*m*_ of pixel position *m* in the image *I*,

(8) $$\begin{array}{c} F_{\text{NLM}}\left(I\left(m\right)\right)=\overset{}{\underset{n\in \Omega_{m}}{\sum }}w\left(m,n\right)I\left(n\right), \end{array}$$

where 0 ≤ *w*(*m*, *n*) ≤ 1,  ∑_*n*∈*Ω*_ *w*(*m*, *n*) = 1. The weights *w*(*m*, *n*) are based on the similarity between the neighbourhoods of pixels *I*(*m*) and *I*(*n*) and are defined as

(9) $$\begin{array}{c} w\left(m,n\right)=\frac{e^{-\left(d\left(N_{m},N_{n}\right)/h^{2}\right)}}{\sum_{n\in I}^{}e^{-\left(d\left(N_{m},N_{n}\right)/h^{2}\right)}}, \end{array}$$

where *N*_*m*_ and *N*_*n*_ are the neighborhoods of the pixel positions *m* and *n*, respectively, *d* is a Gaussian weighted squared Euclidean distance, and *h* is the exponential decay control smoothing parameter. Region *Ω* can be the whole image, but, because of computational reasons, *Ω* uses to be a smaller region in the local neighborhood.

### 2.3. Estimation of **σ**_noise_^2^

Donoho and Johnstone's [19] and Nowak's [20] methods are highly dependent on the noise estimate. We assume that noise distribution, in complex domain, is zero mean Gaussian. Then, noise estimate means noise standard deviation/variance (*σ*_noise_/*σ*_noise_^2^) estimate. In MR images, *σ*_noise_ is usually unknown* a priori* and it must be estimated from the data. A good estimator of *σ*_noise_^2^ of *I* is given in [35, 36] by

(10) $$\begin{array}{c} {\hat{\sigma }}_{\text{noise}}^{2}∶=\frac{2}{4-\pi }\text{Mode}\left\{\sigma_{\text{local}}^{2}\right\}, \end{array}$$

where *σ*_local_^2^ is the local variance of *I*, defined as

(11) $$\begin{array}{c} \sigma_{\text{local}}^{2}\left(m\right)∶=\text{LV}\left(I,m\right), \end{array}$$

where the operator LV is defined in Appendix B.

## 3. A New Probabilistic Wavelet Filter

The wavelet filter proposed in this paper, which will be named hereafter as Villullas-Martin's filter, is defined by the shrinkage

(12) $$\begin{array}{c} F_{\text{VM}}\left(d^{\alpha }\left[k\right]\right)∶=S_{\lambda }^{\alpha }\left[k\right]d^{\alpha }\left[k\right], \end{array}$$

where *d* ^*α*^ are the wavelet coefficients at level/orientation *α* of an image/volume *I* and

(13) $$\begin{array}{c} S_{\lambda }^{\alpha }\left[k\right]∶=\frac{\left(1-\lambda \right)P\left(d^{\alpha }\left[k\right]\;|\;\text{detail}\right)}{\left(1-\lambda \right)P\left(d^{\alpha }\left[k\right]\;|\;\text{detail}\right)+\lambda P\left(d^{\alpha }\left[k\right]\;|\;\text{noise}\right)} \end{array}$$

with parameter *λ* (*S*_*λ*_^*α*^[*k*] represents the posterior probability of being detail given *d* ^*α*^[*k*] with *λ* the prior probability of a coefficient being noise). This filter is based on the filter proposed by Gorgel et al. [26]. The novelty of our filter is the proposed distribution models for details and noise coefficients in the wavelet domain to MRI and the use of the EM method for estimating the parameters. This avoids the problem setting any free parameter such as the noise variance which is usually problematic.

**Figure 2:**
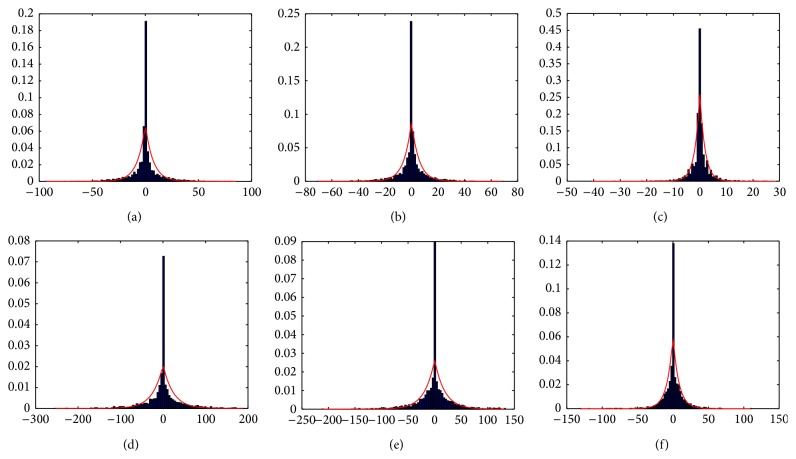
Noiseless image/volume wavelet coefficients (blue bar chart) against approximated Laplacian distribution (red graph).

Noiseless MR images/volumes have a distribution in the wavelet domain with a pronounced maximum at the origin (due to smooth regions) and long tails produced by edges and different structures contained in the image/volume (as shown in Figure 2), so this distribution can be approximately modeled by a Laplacian function, given by

(14) $$\begin{array}{c} P\left(d^{\alpha }\left[k\right]\;|\;\text{detail}\right)∶=\frac{1}{2b}\;e^{-\left(\left|d^{\alpha }\left[k\right]-\mu \right|/b\right)}, \end{array}$$

with parameters *μ* and *b*. This distribution was first proposed to model wavelet coefficients distribution of mammographic images by Gorgel et al. [26].

**Figure 3:**
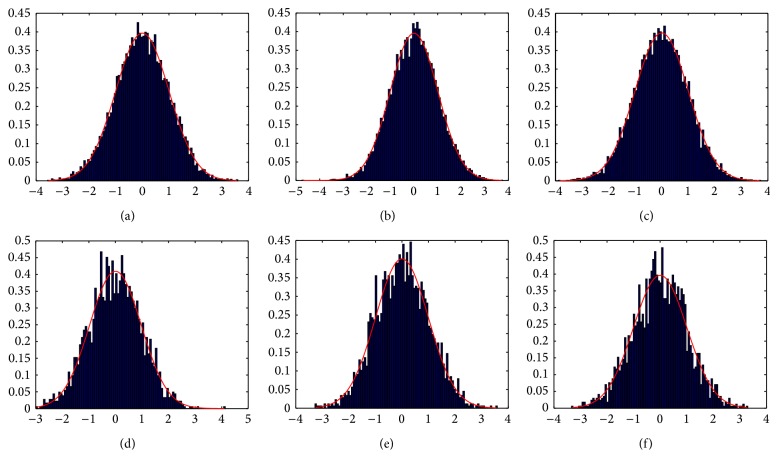
Rayleigh noise image/volume wavelet coefficients (blue bar chart) against approximated Gaussian distribution (red graph).

MR magnitude image/volume distribution is Rician [5, 37]. In high signal to noise ratio (high intensity, bright) regions, Rician distribution tends to a Gaussian distribution and in low signal to noise ratio (low intensity, dark) regions, Rician distribution tends to a Rayleigh distribution [4]; that is, MR noise (signal free) can be modeled by a Rayleigh distribution. In the wavelet domain, the distribution of this noise has a maximum at the origin with short tails (see Figure 3). In this case, we approximate this distribution by a Gaussian distribution, given by

(15) $$\begin{array}{c} P\left(d^{\alpha }\left[k\right]\;|\;\text{noise}\right)∶=\frac{1}{\sigma_{\text{noise}}\sqrt{2\pi }}\;e^{-\left({\left(d^{\alpha }[k]\right)}^{2}/2\sigma_{\text{noise}}^{2}\right)}. \end{array}$$

**Figure 4:**
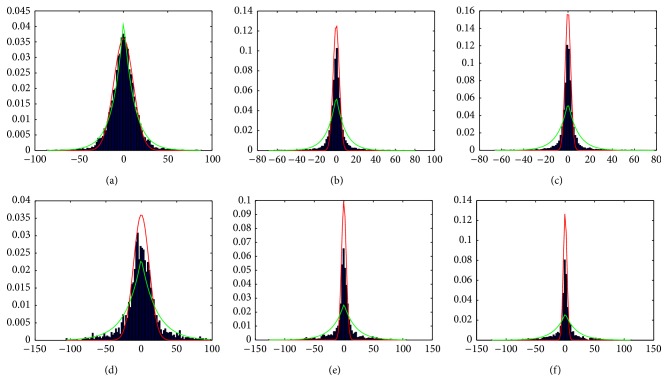
Mixture approximated model distributions (Detail/Laplace = green graph, Noise/Gauss = red graph) superimposed on real image wavelet coefficients histogram (blue bar chart) at level/orientation ∣ NEX: (a) 1/horizontal ∣ 1; (b) 1/horizontal ∣ 10; (c) 1/horizontal ∣ 20; (d) 2/vertical ∣ 1; (e) 2/vertical ∣ 10; (f) 2/vertical ∣ 20.

The definition of *S*_*λ*_^*α*^ as a conditioned probability lets us modify the wavelet coefficients taking into account noise intensity. Besides, the larger image/volume modification by wavelet coefficients shrinkage, the bigger module of the shrunk coefficients; that is, a change in low module wavelet coefficient does not change significantly the image/volume. So, the importance of *S*_*λ*_^*α*^ is focused on the tails of the distributions.  Figure 4 shows the mixture model of detail and noise, with the two approximated distribution models presented before, superimposed on real image/volume wavelet histograms at different levels and orientations.

Parameters estimation in the original paper [26] consider both distributions independently choosing the parameter *λ* depending on the ratio noise/detail. In this paper we consider the joint distribution resulting from considering both distributions allowing us to find the parameters that maximize the similarity between the real and the theoretical distributions. For this purpose, parameters *μ*, *b*, *σ*_noise_, and *λ* are to be calculated by the EM method as Appendix A shows in detail. Here a brief summary is given as follows; given the (independent) known data **X** = {*X*_*i*_}_*i*=1_^*N*^ (vector **X** represents each set of wavelet coefficients *d* ^*α*^ for each scale and orientation *α* of an image/volume. We also drop the upper index *α* for the sake of simplicity along this section.) and the hidden (auxiliary) variables **Z** = {*Z*_*i*_}_*i*=1_^*N*^, defined by

(16) $$\begin{array}{c} Z_{i}∶=\left\{\begin{array}{cc} 1, & \text{if}\;X_{i}\;\text{is}\;\text{detail},\\ 0, & \text{if}\;X_{i}\;\text{is}\;\;\text{noise}, \end{array}\right.\;\;i=1,\ldots ,N, \end{array}$$

the likelihood function to be maximized in this method is

(17) $$\begin{array}{c} L\left(\Theta \;|\;X,Z\right)∶=\overset{N}{\underset{i=1}{\sum }}\ln\left(P\left(X_{i}\;|\;Z_{i},\Theta \right)\right)+\overset{N}{\underset{i=1}{\sum }}\ln\left(P\left(Z_{i}\;|\;\Theta \right)\right), \end{array}$$

where Θ = [*μ*, *b*, *σ*_noise_, *λ*] and *P*(*X*_*i*_∣Θ) = *λP*_Gauss_(*X*_*i*_∣*σ*_noise_)+(1 − *λ*)*P*_Laplace_(*X*_*i*_∣*μ*, *b*), and its expected value is

(18) ELΘ ∣ X,ZΘ,X =−∑i=1Nγiln⁡2+ln⁡b+Xi−μb−ln⁡1−λkkkkkkkkk+1−γi+Xi2σnoise2−ln⁡λ12ln⁡2π+12ln⁡σnoise2+Xi2σnoise2−ln⁡λkkkkkkkkkkkkkkkkkkkk+Xi2σnoise2−ln⁡λ,

with

(19) $$\begin{array}{c} \gamma_{i}=\frac{\left(1-\lambda \right)P_{\text{Laplace}}\left(X_{i}\;|\;\mu ,b\right)}{\left(1-\lambda \right)P_{\text{Laplace}}\left(X_{i}\;|\;\mu ,b\right)+\lambda P_{\text{Gauss}}\left(X_{i}\;|\;\sigma_{\text{noise}}\right)}. \end{array}$$

Maximizing this expression, we obtain

(20) $$\begin{array}{c} \hat{\lambda }∶=1-\frac{1}{N}\overset{N}{\underset{i=1}{\sum }}\gamma_{i};\\ {\hat{\sigma }}_{\text{noise}}^{2}∶=\frac{\sum_{i=1}^{N}\left(1-\gamma_{i}\right)X_{i}^{2}}{\sum_{i=1}^{N}\left(1-\gamma_{i}\right)};\\ \hat{\mu }∶=\arg\underset{\mu =X_{m}}{\min}\overset{N}{\underset{\;i=1}{\sum }}\gamma_{i}\left|\mu -X_{i}\right|;\\ \hat{b}∶=\frac{\sum_{i=1}^{N}\gamma_{i}\left|X_{i}-\hat{\mu }\right|}{\sum_{i=1}^{N}\gamma_{i}}. \end{array}$$

It is an implicit system, so estimators *γ*_*i*_, $\hat{\lambda }$, ${\hat{\sigma }}_{\text{noise}}^{2}$, $\hat{\mu }$, and $\hat{b}$ are calculated by fixed-point iteration with initial conditions

(21) kkklkλ^Ini∶=12,kkklkμ^Ini∶=medianX,kkklkb^Ini∶=1N∑i=1NXi−μ^Ini,σ^noise2Ini∶=medianσlocal2,

where $\sigma_{\text{local}}^{2}∶=\text{LV}(\tilde{X})$ is defined in (B.2) (where $\tilde{X}$ is the vector **X** as a matrix [of wavelet coefficients *d* ^*α*^ at scale and orientation *α*]), and

(22) γiIni∶=1−λ^IniPLaplaceXi ∣ μ^Ini,b^Ini ×1−λ^IniPLaplaceXi ∣ μ^Ini,b^Ini   +λ^IniPGaussXi ∣ σ^noiseIni−1.

## 4. Experiments

In this section, some noisy MR 2D and 3D volumes are filtered to compare the algorithm proposed in this paper with other wavelet filters as well as patch-based and NLM filters found in the reviewed literature and described in Section 2. Section 4.1 contains the description, step by step, of the different filter algorithms applied on the images defined in Section 4.2. The three wavelet methods, proposed by Donoho and Johnstone, Nowak, and Villullas and Martin, apply the approximately shift-invariant scheme, with *K* = 2 in the 2D images and *K* = 1 in the 3D volumes, as proposed in Section 2.1.3, and Donoho-Johnstone's and Nowak's filters use the noise estimator defined in Section 2.3. Patch-based filter, proposed by Awate-Whitaker, uses Gaussian and Rician models (this method can be obtained at the web http://www.itk.org/Doxygen42/html/group_ITKDenoising.html with the predefined parameters values except m_NoiseModel = Gaussian or Rician and m_NoiseModelFidelityWeight = 0.5.), and nonlocal means filter uses predeterminated parameters (this method can be obtained at the web http://www.mathworks.com/matlabcentral/fileexchange/40162-james-stein-type-center-pixel-weights-for-non-local-means) with the same noise estimator as Nowak's and Donoho-Johnstone's filters. The three wavelet filters and NLM filter have been programming in MATLAB and Awate-Whitaker's filter has been programmed in C.

### 4.1. Filter Algorithms

The different filtering methods used in experiments shown in Section 4.4 are next described step by step for a given image/volume *I*. The wavelet used in these experiments is the Haar wavelet. As the Haar wavelet support is minimal and a wavelet coefficient image/volume is a windowed weighted average of an image/volume, this wavelet seems the most logical choice when we want to remove noise and preserve details in an image/volume filtering, against other wavelet families with larger supports.

#### 4.1.1. Donoho-Johnstone's Algorithm

(i) Calculate noise variance estimator ${\hat{\sigma }}_{\text{noise}}^{2}$ as Section 2.3 shows.
(ii) Compute the (*J* = 2)-scale DWT of the magnitude image/volume *I*.
(iii) Filter the wavelet coefficients *d* ^*α*^ through the Donoho-Johnstone's filter, *F*_DJ_, defined in Section 2.1.
(iv) Compute the inverse DWT of the filtered wavelet and scaling coefficients to obtain an estimate of denoised image/volume.
(v) Repeat Steps (ii)–(iv), with the image/volume shifted {−*K*,…, −1,0, 1,…, *K*} pixels/voxels in each direction, as Section 2.1.3 explains, and average all shifted denoised images/volumes (original image/volume *I* corresponds to null shift).

#### 4.1.2. Nowak's Algorithm

(i) Calculate noise variance estimator ${\hat{\sigma }}_{\text{noise}}^{2}$ as Section 2.3 shows.
(ii) Compute the (*J* = 2)-scale DWT of the squared magnitude image/volume *I* ^2^.
(iii) Remove the bias from the scaling coefficients *s* ^*J*^ by subtracting *C* = 2^*J*+1^ *σ*_noise_ from each (see [20]).
(iv) Filter the wavelet coefficients *d* ^*α*^ through the Nowak's filter, *F*_N_, defined in Section 2.1.
(v) Compute the inverse DWT of the filtered wavelet and unbiased scaling coefficients to obtain an estimate of squared denoised image/volume.
(vi) Take the pixel-by-pixel/voxel-by-voxel square-root of the result to obtain an estimate of the unbiased image/volume.
(vii) Repeat Steps (ii)–(vi), with the image/volume shifted {−*K*,…, −1,0, 1,…, *K*} pixels/voxels in each direction, as Section 2.1.3 explains, and average all shifted denoised images/volumes (original image/volume *I* corresponds to null shift).

#### 4.1.3. Awate-Whitaker's Algorithm

(i) Let *I*_0_ = *I* the noisy image/volume and *k* > 0.
(ii) For each region ${\hat{z}}^{k}=[{\hat{x}}^{k},{\hat{y}}^{k}]$ (i.e., for each pixel *m*) of the image/volume *I*_*k*_, compute $\partial h(\hat{X}|\hat{Y}={\hat{y}}^{k})/\partial {\hat{x}}^{k}$.
(iii) Use the descending gradient differences to calculate ${\hat{x}}^{k+1}$ by ${\hat{x}}^{k+1}={\hat{x}}^{k}-\delta \left(\partial h/\partial {\hat{x}}^{k}\right)$.
(iv) The image/volume *I*_*k*+1_ is given by the intensity ${\hat{x}}^{k+1}$ at pixel *t*. If *k* + 1 is less than the maximum number of iterations, go to Step (ii) with *k* = *k* + 1. Otherwise, the filtered image/volume is *I*_*k*+1_.

#### 4.1.4. Nonlocal Means Algorithm

(i) Given the parameters values, calculate the weights function *w* for each pixel of the image/volume *I*.
(ii) For each pixel, compute the NLM filtered image/volume with the weight function.

#### 4.1.5. Villullas-Martin's Algorithm

(i) Compute the (*J* = 2)-scale DWT of the magnitude image/volume *I*.
(ii) Filter the wavelet coefficients *d* ^*α*^ through the Villullas-Martin's filter, *F*_VM_, defined in Section 3.
(iii) Compute the inverse DWT of the filtered wavelet and scaling coefficients to obtain an estimate of the denoised image/volume.
(iv) Repeat Steps (i)–(iii), with the image/volume shifted {−*K*,…, −1,0, 1,…, *K*} pixels/voxels in each direction, as Section 2.1.3 explains, and average all shifted denoised images/volumes (original image/volume *I* corresponds to null shift).

### 4.2. MR Data Sets

The experiments were conducted on fifth MRI data sets. The first and second data sets consist of simulated MR volumes and images obtained from the Brainweb database [38]. The third and fourth data sets were collected from Centro de Diagnóstico Valladolid (CDV) QDIAGNOSTICA in Valladolid (Spain). The last data set was obtained from a database of the Laboratory of Image Processing (LPI) of the University of  Valladolid. Details about these data sets are described in following subsections.

#### 4.2.1. Simulated MR Images

Simulated MR images/volumes are a useful data set which allows a first evaluation of different analysis methods. In the 3D case, the noiseless image volume consists of a 3-dimensional volume of resolution 180 × 216 × 4 extracted from a volume generated in the Brainweb database of resolution 181 × 217 × 181, T1-weighted, 1 mm slice thickness, 0% of noise, and RF = 0%. Image intensity values vary in {0,1,…, 255}. In the 2D case, the noiseless image consists of a 2-dimensional axial section of the 3-dimensional volume of resolution 180 × 216. Rician noise was generated by the equation

(23) $$\begin{array}{c} \hat{I}∶=\sqrt{{\left(I+N_{1}\right)}^{2}+N_{2}^{2}}, \end{array}$$

where *I* is the noiseless MR image/volume and *N*_*k*_/*k* ∈ {1,2} are *N*(0, *σ* ^2^) independent identically distributed Gaussian random variables, with *σ* ∈ {5,6,…, 20}.

#### 4.2.2. Real MR Images

Real data set consists of two data sets. Real data images are 2D axial section of a brain, acquired using a General Electric Signa 1.5 T scanner, T1-weighted, and 0.9375 × 0.9375 mm^2^ pixel size for both data sets. The first data set is composed of 4 images of dimension 256 × 256, varying NEX ∈{4,8, 16,64}, TR = 6 ms, TE = 1.588 ms, and flip angle = 15°. The second data set consists of 32 images with dimension 192 × 160, NEX = 1, TR = 40 ms, TE = 9 ms, and flip angle = 90°. As noiseless image cannot be obtained for real data, noiseless image approximations are given by the following: in the first data set, the noiseless image approximation corresponds to NEX = 64; in the second data set, the noiseless image approximation is the average in the complex domain of the 32 images that this set consists of.

#### 4.2.3. Clinical MR Images

Real clinical data set consists of 20 MR T1-weighted acquisitions from different subjects. The dataset was acquired in a General Electric Signa 1.5 T scanner with NEX = 1, 2.1875 × 2.1875 mm^2^ pixel size, and 256 × 256 image dimension. Other parameters values are TR = 5.8020 ms, TE = 1.7280 ms, and flip angle = 10°. This set of images try to evaluate the different filter methods with images taken from different subjects (anatomy variations). In this case, as no ground truth is available, the evaluation will be performed based on experts' rankings.

### 4.3. Quality Measures

After filtering these images/volumes (*I* is the noiseless image/volume, *I*_*N*_ is the noisy image/volume, and *I*_*F*_ is the filtered noisy image/volume), the results obtained are compared using the following efficiency measurements (see Appendix B for details).

#### 4.3.1. Averaged Error Local Variance (AELV)

AELV is an objective quality measure [39] that quantifies the deviation of estimated values from the true value. Specifically, the AELV of *I*_*F*_ with respect to *I* is measured as

(24) $$\begin{array}{c} \text{AELV}\left(I_{F},I\right)∶=\frac{1}{M}\underset{m}{\sum }\text{LV}\left(I-I_{F},m\right), \end{array}$$

where *M* is the total number of pixels/voxels of *I* and the operator LV is defined in Appendix B.

#### 4.3.2. Normalized Averaged Error Local Variance (NAELV)

Variations in AELV can be difficult to understand. To test at what rate reduces its value after filtering, AELV normalization is used as

(25) $$\begin{array}{c} \text{NAELV}\left(I_{F},I_{N},I\right)∶=\frac{\text{AELV}\left(I_{F},I\right)}{\text{AELV}\left(I_{N},I\right)}. \end{array}$$

#### 4.3.3. Structural Similarity (SSIM)

Although AELV is a useful measure of similarity, it is not suitable to obtain a comparison similar to that performed by the human eye [40, 41]. Most common alternative is SSIM which is consistent with the visual perception. The SSIM index is estimated as

(26) SSIMIF,I∶=2GMIGMIF+C1GM2I+GM2IF+C1 ·2GCVI,IF+C2GVI+GVIF+C2,

where the operators GM(*I*), GV(*I*), and GCV(*I*, *I*_*F*_) are defined in Appendix B, *C*_1_∶ = 6.5025 and *C*_2_∶ = 58.5225.

#### 4.3.4. Averaged Local Signal to Noise Ratio (ALSNR)

Another simple method of checking the noise level in an image is the averaged local SNR, measured as

(27) $$\begin{array}{c} \text{ALSNR}\left(I_{F},I\right)∶=\frac{1}{M}\overset{}{\underset{m}{\sum }}\frac{\text{LV}\left(I,m\right)}{\text{LV}\left(I-I_{F},m\right)}, \end{array}$$

where *M* is the total number of pixels/voxels of *I* and the operator LV is defined in Appendix B.

#### 4.3.5. Contrast

**Figure 5:**
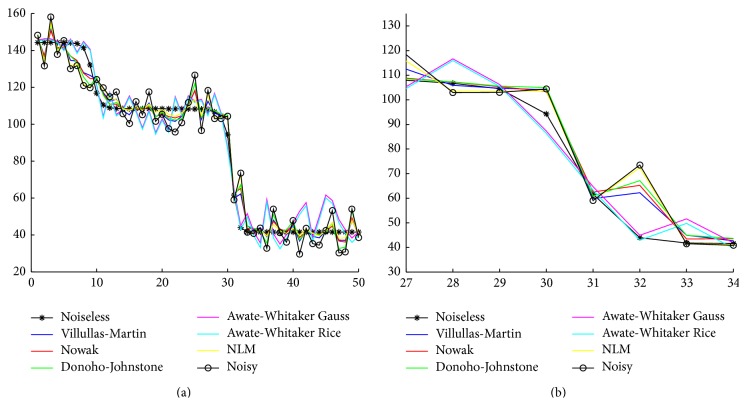
Comparative profiles of 1D section of noiseless, noisy, and the different filtered 2D images. (a) 1D section of Section 4.2.1 data set image with parameter *σ* = 10 from pixel (50,126) to pixel (99,126); (b) detail of profile (a) in coordinates [27,28,…, 34].

The measure normally used to calculate images contrast is given by

(28) $$\begin{array}{c} \text{Cont}\left(I\right)∶=\frac{S_{\max}-S_{\min}}{S_{\max}+S_{\min}}, \end{array}$$

where *S*_max⁡_ and *S*_min⁡_ are the maximum and minimum value in a specific ROI of the image/volume *I*. This quality measurement is not effective enough because some filters (e.g., some wavelet filters) generate a bias that modifies the values of this quality measurement but does not affect the contrast of the image/volume. Besides, there are many tools, used by radiologists, which let them modify the window/level of the image/volume. We can observe the variation of contrast taking a section of the corresponding images/volumes to compare the different changes of intensity.  Figure 5 shows that wavelet filters preserve edge changes of intensity very well due to its property of locality.

### 4.4. Numerical Experiments

In these experiments, the size of a neighborhood at pixel/voxel *m* (on the image/volume *I*), size (*N*_*m*_), used is size (*N*_*m*_) = 5 × 5 (|*N*_*m*_ | = 25) in 2-dimensional case and size (*N*_*m*_) = 5 × 5 × 5 (|*N*_*m*_ | = 125) in 3-dimensional case in (B.2) and (B.1) in Appendix B. Others values give us different measurement values but the same visual distribution.

#### 4.4.1. Experiment 1: Filtering Simulated Data

**Figure 6:**
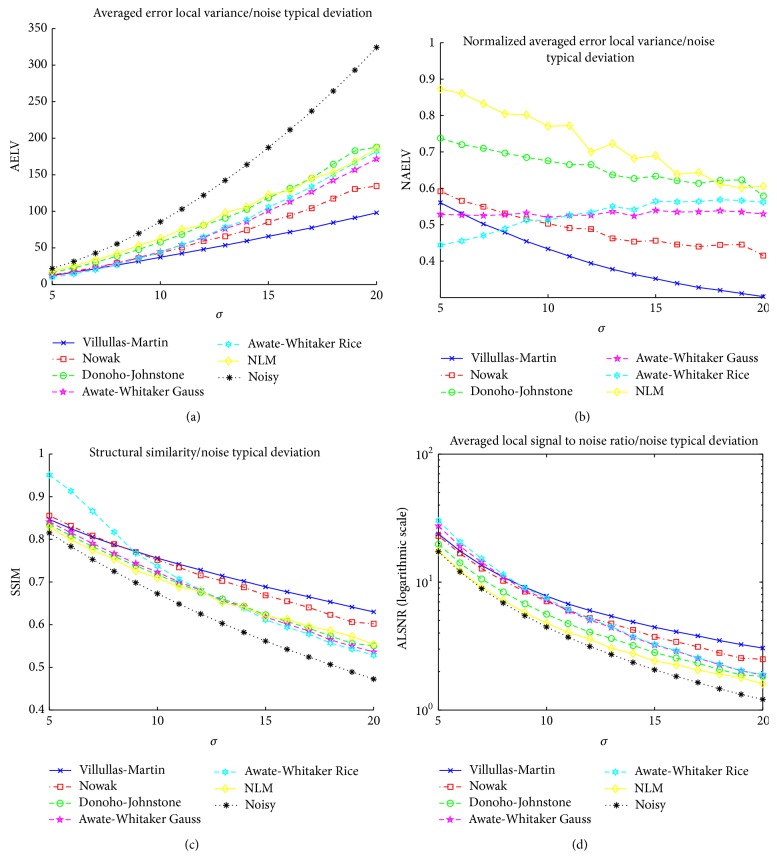
Comparative results for the different filtering methods in experiment 1 with parameter *σ* ∈ {5,6,…, 20}.

**Figure 7:**
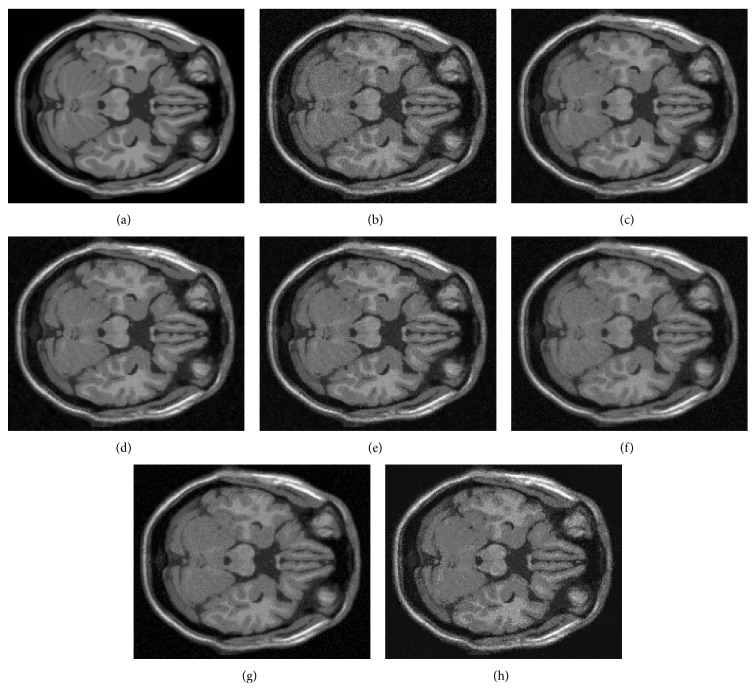
Example of experiment 1 with parameter *σ* = 15. (a) Noiseless image; (b) noisy image; (c) noisy image filtered by Villullas-Martin's method; (d) noisy image filtered by Nowak's method; (e) noisy image filtered by Donoho-Johnstone's method; (f) noisy image filtered by Awate-Whitaker's method with Gaussian model; (g) noisy image filtered by Awate-Whitaker's method with Rician model; (h) noisy image filtered by nonlocal means method.

In the first experiment, the 16 2D simulated data sets described in Section 4.2.1 are filtered. The parameter values used to generate noisy images are *σ* ∈ {5,6,…, 20}.  Figure 6 shows the comparison of the different averaged quality measurements as function of the parameter *σ* (each parameter *σ* has associated 20 simulated images experiments and the corresponding quality measurement value is the average of the 20 quality measurement values of the corresponding images. We use this 20 data sets to reduce the variability of the quality measurements). For low values of the parameter *σ* (almost noiseless images), Awate-Whitaker's filter (Rician over Gaussian in SSIM measurement and Gaussian over Rician in AELV measurement) have a good noise removal and it beats the other methods in this case. Nowak's filter gets results close to Villullas-Martin's filter (in fact, Nowak's filter improves Villullas-Martin filter in SSIM for tiny values of *σ*) but as parameter value increases, difference between Villullas-Martin's filter and Nowak's filter grows. Villullas-Martin's filter is clearly the best method for medium and high values of *σ* (hard noisy images. This value corresponds to low NEX in image acquisition). NAELV graph shows us that Awate-Whitaker's filter has constant improvement proportion in contrast to wavelet filters, which amplify its improvement proportion with *σ*. Then, Awate-Whitaker's filter is a good denoising method for very low values of *σ* but as *σ* increases, the filter's strength decreases. The NLM filter has a behavior near to Nowak's filter, better than Nowak's filter in high values of *σ* and worst in low and medium values of *σ*.  Figure 7 shows an example (*σ* = 15) where visual differences between the filtering methods can be seen. In this case, Villullas-Martin's filtering and Nowak's filtering are similar to each other and better than Donoho-Johnstone's filtering which has lower noise removal. NLM filter is the worst choice because the noise has not been properly eliminated inside brain structure.

#### 4.4.2. Experiment 2: Filtering Real Data

**Figure 8:**
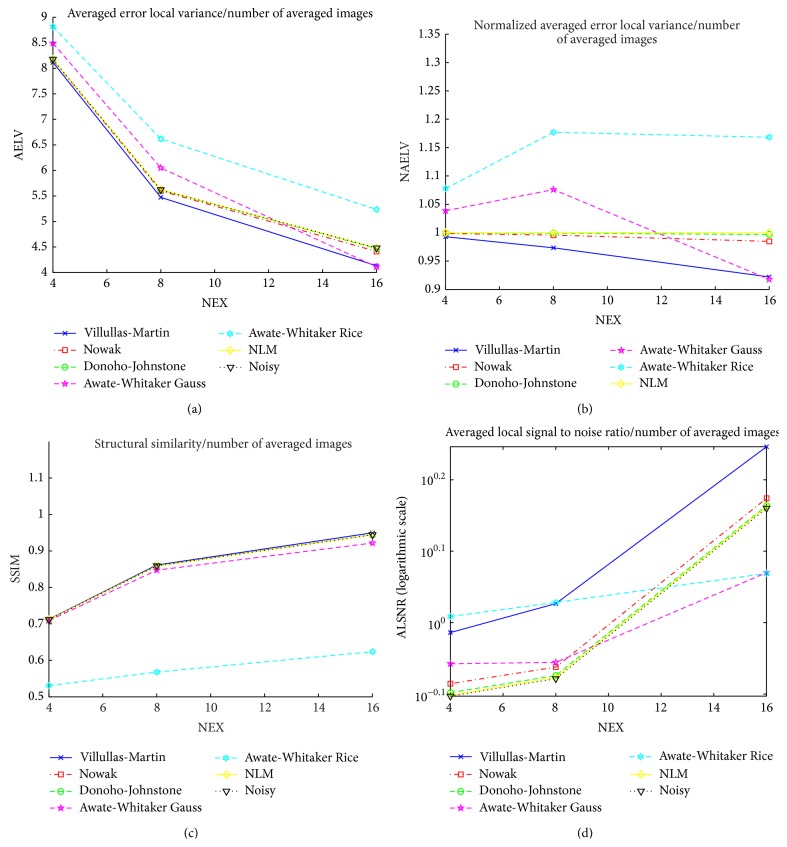
Comparative results for the different filtering methods in experiment 2 with number of averaged images NEX ∈{4,8, 16}.

**Figure 9:**
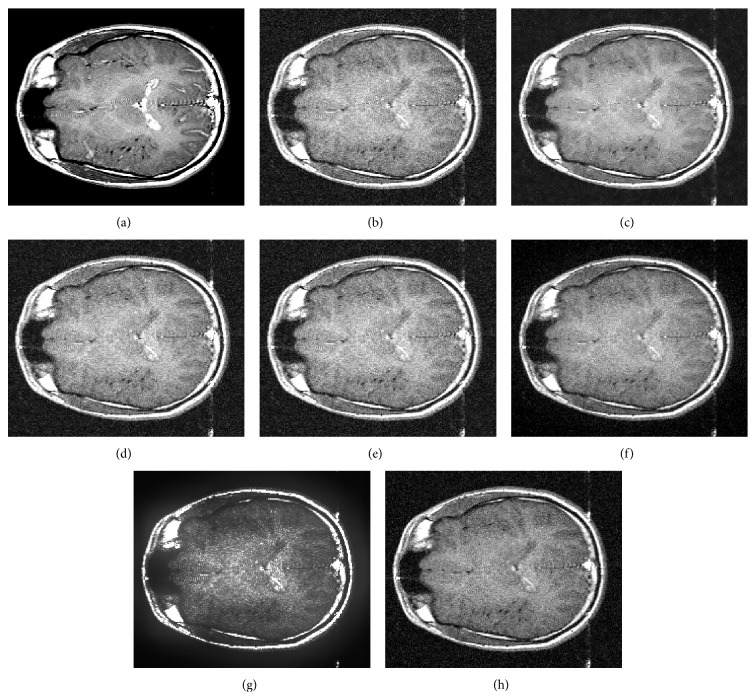
Example of experiment 2 with number of averaged images NEX = 8. (a) Noiseless image; (b) noisy image; (c) noisy image filtered by Villullas-Martin's method; (d) noisy image filtered by Nowak's method; (e) noisy image filtered by Donoho-Johnstone's method; (f) noisy image filtered by Awate-Whitaker's method with Gaussian model; (g) noisy image filtered by Awate-Whitaker's method with Rician model; (h) noisy image filtered by nonlocal means method.

In the second experiment, we have filtered the first real data set described in Section 4.2.2. Noisy images set is the images belonging to NEX ∈{4,8, 16} and noiseless image is generated with NEX = 64.  Figure 8 shows the comparisons of the different quality measurements as function of NEX. It can be seen that Villullas-Martin's filter improves Nowak's and Donoho-Johnstone's filters, with a slight increase as NEX increase. Awate-Whitaker's filters cannot improve wavelet filters results (moreover, as AELV graph shows, Awate-Whitaker's filter ruins low NEX images). As Experiment 1, NLM filter works as good as Nowak's filter.  Figure 9 shows the example of NEX = 8. In this example we can see that Villullas-Martin's filter provide a better filtering, where more noise is removed than Nowak's, Donoho-Johnstone's, and NLM filters. Awate-Whitaker's filter with Gaussian model obtains a bit worse denoising than Nowak's, Donoho-Johnstone's, and NLM filters and Awate-Whitaker's filter with Rice model does not remove enough noise inside brain structures and generates artifacts.

#### 4.4.3. Experiment 3: 3D Wavelet Filtering

**Figure 10:**
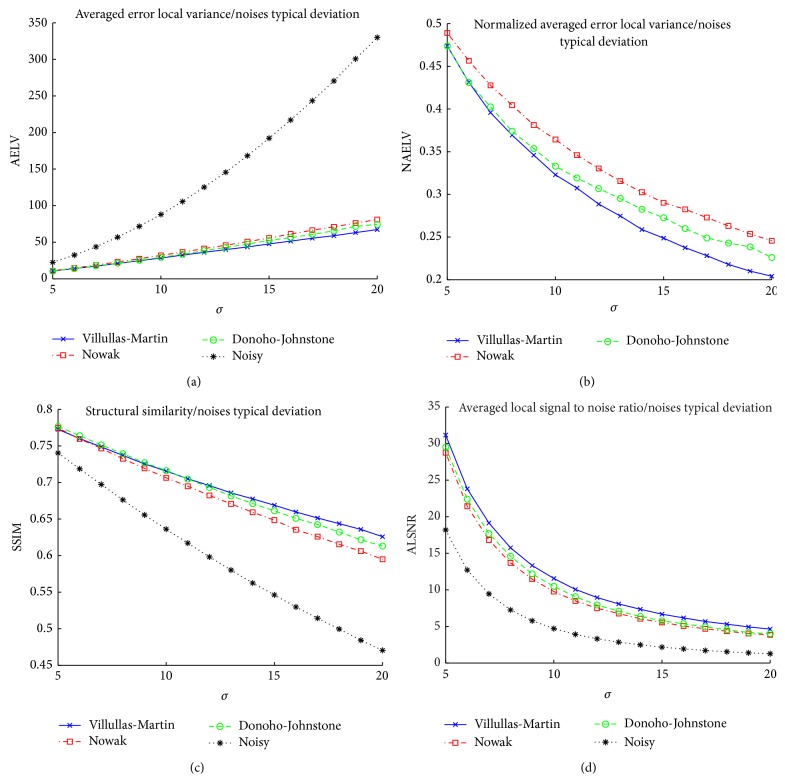
Comparative results for the different filtering methods in experiment 3 with parameter *σ* ∈ {5,6,…, 20}.

This experiment shows the strength of the wavelet filters in the 3-dimensional case. In this case, the 16 3D simulated data volumes described in Section 4.2.1 for *σ* ∈ {5,6,…, 20} are filtered.  Figure 10 shows the comparison of the different averaged (20 volumes for each *σ* level whose quality measurements are averaged for each *σ*) quality measurements as function of *σ*. This experiment shows the same behavior as Experiment 1 in wavelet filters. All of these methods improve the filtering in 3D case preserving the relationship between them.

#### 4.4.4. Experiment 4: Filtered Real Image against Higher NEX

**Figure 11:**
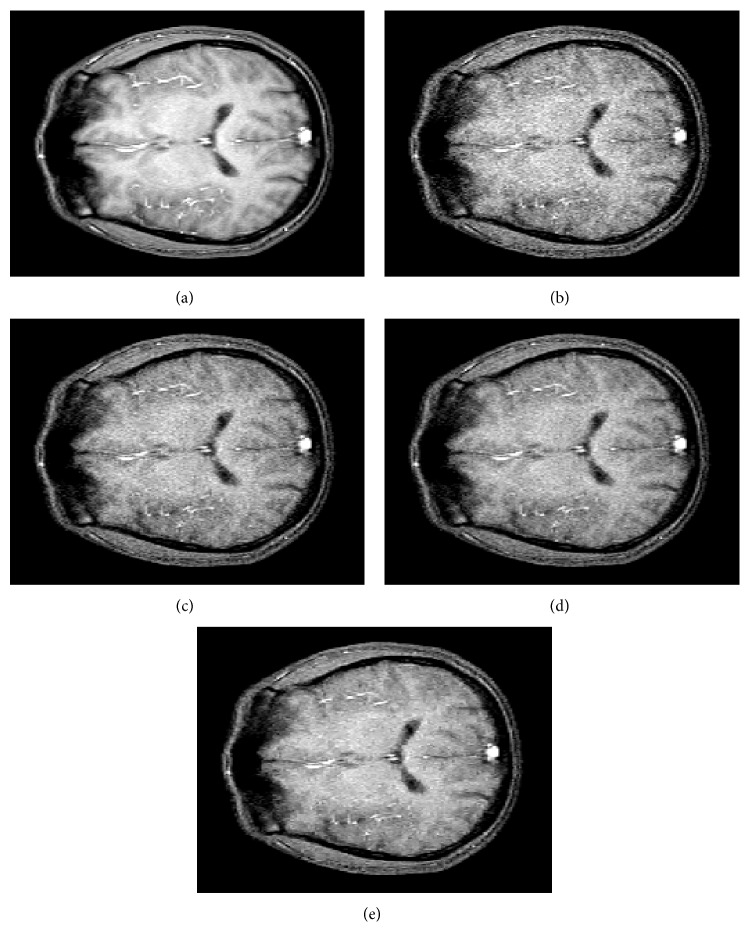
Experiment 4. (a) Noiseless image; (b) noisy image for NEX = 2; (c) noisy image for NEX = 3; (d) noisy image for NEX = 4; (e) noisy image for NEX = 2 filtered by Villullas-Martin method.

The forth experiment evaluates the filtering power of Villullas-Martin's filter. This filter is compared with NEX rising. For this comparison, the second real data set described in Section 4.2.2 is used. The “same” 32 images with NEX = 1 let us control noise level, which can be reduced by averaging *N* images in the complex domain, where higher values of *N* imply lower noise level. The selected image to be filtered belongs to *N* = 2.  Table 1 shows that the first value of averaged images (*N*) which improves the quality measurements of filtered image with *N* = 2 is *N* = 4; that is, Villullas-Martin's filter lets us obtain images with quality as good as with double acquisition time.  Figure 11 shows the images involved in this experiment. It can be seen that filtered image with *N* = 2 has less noise at smooth regions inside the structure than the image with no filtering with *N* = 3.

#### 4.4.5. Experiment 5: Clinical Experts Comparison

**Figure 12:**
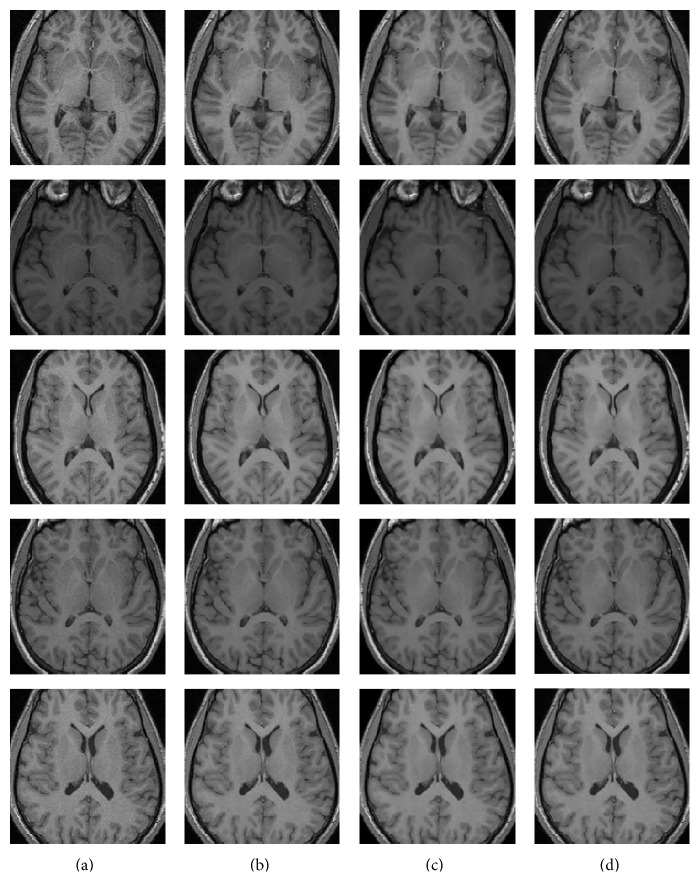
Examples subset of experiment 5. (a) Noisy images; (b) noisy images filtered by Villullas-Martin's method; (c) noisy images filtered by Nowak's method; (d) noisy images filtered by Donoho-Johnstone's method.

In the last experiment, data set images of Section 4.2.3 are filtered by the wavelet filter methods described in Section 2.1. The goal of this experiment is to evaluate the clinical relevance of the proposed filter over other wavelet filters with the help of 3 radiology experts at high SNR images (where the three wavelet filters are more similar). The images are filtered with Villullas-Martin's, Nowak's, and Donoho-Johnstone's filter methods, which have proven quite efficient in previous experiments. The experts rank the filtered images by their visual quality based on their experience (1 = best, 2 = medium, and 3 = worst). The criteria to evaluate this quality are based on the ease of recognizing different structures in the image (each expert uses different regions that he judges to be key). The ranking is blind in the sense that the filtering method used to filter each image is hidden to the expert. To evaluate the intraexpert variability the experiment was repeated during two different days. Tables 2 and 3 show results achieved for the intraexpert and interexpert variability, respectively. Although the Villullas-Martin's filter does not remove as much noise as other filters (apparently), which obtains similar filtering results in these cases, the result image given by our method was considered by the experts visually nicer and with higher contrast in the boundaries of the image, which eases radiologist work in identifying the different brain structures; that is, Villullas-Martin's filter preserves structures better than the other wavelet filters after denoising. The experiment results can be summarized as follows. In 84.2% of cases, Villullas-Martin's filter was chosen as the best filter method (rank = 1), whereas Nowak's filter was chosen as the worst filter in 88.3% of cases (rank = 3). Donoho-Johnstone's filter was chosen as the medium filter (rank = 2) in 75% of cases.  Table 4 shows all percentages.  Figure 12 shows some example images in this experiment.

## 5. Conclusion

A new wavelet-domain filtering has been proposed in this paper. Assuming that wavelet coefficients of a noiseless MR image/volume can be modeled by a Laplacian distribution (as we know in brain, the filter can be adapted to other human parts easily with minor or no changes), and the wavelet coefficients distribution of Rayleigh noise can be approximated by a Gaussian distribution, a probabilistic method has been proposed; that is, wavelet coefficients are shrunk depending on its conditioned probability of being noise or detail (posterior probability). To calculate the parameters involved in the expression of  *F*_VM_, EM method has been used. This fact makes a filter independent of *σ*_noise_ estimators, in contrast to other methods like Donoho-Johnstone's and Nowak's.  Section 4 shows that Villullas-Martin's filter can provide better noise removal than Nowak's, Donoho-Johnstone's, Awate-Whitaker's, and nonlocal means filters for several signal to noise ratios and let us obtain high quality images/volumes with lower acquisition time, as much in 2D images as 3D volumes. In addition, a clinical evaluation performed by 3 radiology experts shows that our filter clearly outperforms the others. This experiment allows us to state that apparently to eliminate more noise not always means the visual quality expected by the experts is better, because this noise removal could imply low contour conservation and less details. Our filter result seems to the experts more similar to what they expect to see to better identify anatomical structures based on their experience. In addition, F_VM_ can be faster than *F*_N_ due to the expensive computation of ∑_*m*_(*ψ* ^*α*^)^2^[*k*](*m*)*I* ^2^(*m*) at $\hat{{\left(\sigma^{\alpha }\right)}^{2}[k]}$ estimator for the case of a general wavelet (see Section 2.1 and [20]).

As future research lines, we plan to generalize the distribution models of the wavelet coefficients both for the details and for the noise. For the details a good candidate will be the generalized Gaussian distribution which seems very promising. In addition to that, we plan to check whether the proposed method can be also applied to other body parts as well as to other MR modern modalities such as fast, ultrafast, and low-field MRI for which the noise level is known to be very high.

## Figures and Tables

**Table 1:** Measurement results for NEX = 2, 3, 4 and Villullas-Martin's method for NEX = 2.

Image∖measurement	AELV	SSIM	ALSNR
NEX = 2	60.5142	0.6355	2.1124
NEX = 3	39.2910	0.7162	3.2623
NEX = 4	28.6274	0.7698	4.5433
NEX = 2 filtered	30.5097	0.7331	3.4963

**Table 2:** Intraexpert evaluation percentages. Success implies that the expert chooses the same quality rank for the same subject for the two different instants of time; fail implies that the expert chooses different quality rank for the same subject.

	Expert 1	Expert 2	Expert 3
Success	95%	60%	70%
Fail	5%	40%	30%

**Table 3:** Interexpert evaluation percentages. Success implies that the experts choose the same quality rank for the same subject for the two different instants of time; fail implies that the experts choose different quality rank for the same subject. In these results only intraexpert coincidences are taken into account.

	Experts 1 and 2	Experts 1 and 3	Experts 2 and 3
Success	75%	85%	65%
Fail	25%	15%	35%

**Table 4:** Total percentages in all of the experiment images used in experiment 5. The different wavelet filters were ranked by 3 radiology experts according to their visual quality in two different instants of time.

	Villullas-Martin	Nowak	Donoho-Johnstone
Best	84.2%	0.8%	15%
Middle	14.1%	10.9%	75%
Worst	1.7%	88.3%	10%

## Appendices

### A. E.M. Parameter Estimation

We estimate the filter parameters by the EM method. This appendix shows the detailed derivation of the estimation equations.

Let Θ = [*μ*, *b*, *σ*_noise_, *λ*] be the unknown parameter vector, **X** = {*X*_*i*_}_*i*=1_^*N*^ the known data, and **Z** = {*Z*_*i*_}_*i*=1_^*N*^ hidden auxiliary variables defined by

(A.1) $$\begin{array}{c} Z_{i}∶=\left\{\begin{array}{cc} 1, & \text{if}\;X_{i}\;\;\text{is}\;\;\text{detail},\\ 0, & \text{if}\;X_{i}\;\text{is}\;\text{noise}, \end{array}\right.\;\;i=1,\ldots ,N. \end{array}$$

We assume that **X** = {*X*_*i*_}_*i*=1_^*N*^ are independent. So

(A.2) PXi ∣ Θ=λPGaussXi ∣ σnoise +1−λPLaplaceXi ∣ μ,b,PX ∣ Θ=∏i=1NPXi ∣ Θ.

Including hidden variables,

(A.3) $$\begin{array}{c} P\left(X,Z\;|\;\Theta \right)=\overset{N}{\underset{i=1}{\prod }}P\left(X_{i},Z_{i}\;|\;\Theta \right). \end{array}$$

Moreover,

(A.4) $$\begin{array}{c} P\left(X_{i},Z_{i}\;|\;\Theta \right)=P\left(X_{i}\;|\;Z_{i},\Theta \right)P\left(Z_{i}\;|\;\Theta \right), \end{array}$$

so

(A.5) PXi ∣ Θ=PXi ∣ Zi=1,ΘPZi=1 ∣ Θ +PXi ∣ Zi=0,ΘPZi=0 ∣ Θ=PLaplaceXi ∣ μ,b1−λ +PGaussXi ∣ σnoiseλ.

We define the likelihood function that we optimize as

(A.6) LΘ ∣ X,Z∶=ln⁡PX,Z ∣ Θ=∑i=1Nln⁡PXi,Zi ∣ Θ=∑i=1Nln⁡PXi ∣ Zi,Θ+∑i=1Nln⁡PZi ∣ Θ.

The expected value of this function is

(A.7) ELΘ ∣ X,ZΘ,X =E∑i=1Nln⁡PXi ∣ Zi,Θ+∑i=1Nln⁡PZi ∣ ΘΘ,X =∑i=1NEln⁡PXi ∣ Zi,ΘΘ,X  +∑i=1NEln⁡PZi ∣ ΘΘ,X =∑i=1NEln⁡PXi ∣ Zi,ΘΘ,Xi  +∑i=1NEln⁡PZi ∣ ΘΘ,Xi =∑i=1NPZi=1 ∣ Θ,Xiln⁡PXi ∣ Zi=1,Θ    +PZi=0 ∣ Θ,Xiln⁡⁡PXi ∣ Zi=0,Θ    +PZi=1 ∣ Θ,Xiln⁡⁡PZi=1 ∣ Θ    +PZi=0 ∣ Θ,Xiln⁡PZi=0 ∣ Θ.

Let *γ*_*i*_∶ = *P*(*Z*_*i*_ = 1∣Θ, *X*_*i*_) (then *P*(*Z*_*i*_ = 0∣Θ, *X*_*i*_) = 1 − *γ*_*i*_), so

(A.8) ELΘ ∣ X,ZΘ,X =∑i=1Nγiln⁡PLaplaceXi ∣ μ,b    +1−γiln⁡PGaussXi ∣ σnoise    +PLaplaceXiμ,bγiln⁡1−λ+1−γiln⁡λPLaplaceXi ∣ μ,b =∑i=1Nγiln⁡PLaplaceXi ∣ μ,b+ln⁡1−λ    +1−γiln⁡PGaussXi ∣ σnoise+ln⁡λln⁡PLaplaceXi ∣ μ,b+ln⁡1−λ.

As

(A.9) γi=PZi=1 ∣ Θ,Xi=PXi ∣ Zi=1,ΘPZi=1 ∣ ΘPXi ∣ Θ=PXi ∣ Zi=1,ΘPZi=1 ∣ Θ ×PXi ∣ Zi=1,ΘPZi=1 ∣ Θ  k+PXi ∣ Zi=0,ΘPZi=0 ∣ Θ−1,

then

(A.10) $$\begin{array}{c} \gamma_{i}=\frac{\left(1-\lambda \right)P_{\text{Laplace}}\left(X_{i}\;|\;\mu ,b\right)}{\left(1-\lambda \right)P_{\text{Laplace}}\left(X_{i}\;|\;\mu ,b\right)+\lambda P_{\text{Gaus}s}\left(X_{i}\;|\;\sigma_{\text{noise}}\right)}. \end{array}$$

Replacing *P*_Laplace_ *y* *P*_Gauss_ at expected value expression

(A.11) ELΘ ∣ X,ZΘ,X =−∑i=1Nγiln⁡⁡2+ln⁡⁡b+Xi−μb−ln⁡⁡1−λ     +1−γi+Xi2σnoise212ln⁡⁡2π+12ln⁡⁡σnoise2          + Xi2σnoise2−ln⁡⁡λ.

To maximize this function, we calculate the gradient and make it equal to 0:

(A.12) 0=∂∂λELΘ ∣ X,ZΘ,X=∑i=1Nγi−11−λ+1−γi1λ=∑i=1N1−λ1−γi−λγi1−λλ.

Therefore

(A.13) λ^∶=1−1N∑i=1Nγi,0=∂∂σnoise2ELΘ ∣ X,ZΘ,X=∑i=1N1−γi12σnoise2−Xi22σnoise4.

Therefore

(A.14) $$\begin{array}{c} {\hat{\sigma }}_{\text{noise}}^{2}∶=\frac{\sum_{i=1}^{N}\left(1-\gamma_{i}\right)X_{i}^{2}}{\sum_{i=1}^{N}\left(1-\gamma_{i}\right)}. \end{array}$$

The estimator of *μ*, $\hat{\mu }$ cannot be determined using this technique as the expression ∑_*i*=1_^*N*^ *γ*_*i*_ | ·−*X*_*i*_| is piecewise-linear and its minimum is always in a vertex. So in this case a direct method is proposed:

(A.15) $$\begin{array}{c} \hat{\mu }∶=\arg\underset{\mu =X_{m}}{\min}\;\overset{N}{\underset{i=1}{\sum }}\gamma_{i}\left|\mu -X_{i}\right|. \end{array}$$

Finally the estimation of the shape parameter *b* is given by

(A.16) $$\begin{array}{c} 0=\frac{\partial }{\partial b}E\left(L{\left.\left(\Theta \;|\;X,Z\right)\right|}_{\Theta ,X}\right)=\overset{N}{\underset{i=1}{\sum }}\gamma_{i}\left(\frac{1}{b}-\frac{\left|X_{i}-\mu \right|}{b^{2}}\right). \end{array}$$

Therefore

(A.17) $$\begin{array}{c} \hat{b}∶=\frac{\sum_{i=1}^{N}\gamma_{i}\left|X_{i}-\hat{\mu }\right|}{\sum_{i=1}^{N}\gamma_{i}}. \end{array}$$

### B. Measurements

This appendix contains different functions used along the paper. The function |·| is the cardinal of the corresponding set (|*A* | = Cardinal of the set *A*).

(i) The local mean of an image/volume *I* at pixel/voxel position *m* is defined as
  (B.1) $$\begin{array}{c} \text{LM}\left(I,m\right)∶=\frac{1}{\left|N_{m}\right|}\underset{i\in N_{m}}{\sum }I\left(i\right), \end{array}$$
where *N*_*m*_ is a neighborhood of pixel/voxel *m*.
(ii) The local variance of an image/volume *I* at pixel/voxel position *m* is defined as
  (B.2) $$\begin{array}{c} \text{LV}\left(I,m\right)∶=\frac{1}{\left|N_{m}\right|}\underset{i\in N_{m}}{\sum }I^{2}\left(i\right)-\text{LM}{\left(I,m\right)}^{2}, \end{array}$$
where *N*_*m*_ is a neighborhood of pixel/voxel *m*.
(iii) The global mean of an image/volume *I* is defined as
  (B.3) $$\begin{array}{c} \text{GM}\left(I\right)∶=\frac{1}{M}\underset{m}{\sum }I\left(m\right), \end{array}$$
where *M* is the total number of pixels/voxels and *m* represents the different pixel/voxel positions on the image/volume *I*.
(iv) The global variance of an image/volume *I* is defined as
  (B.4) $$\begin{array}{c} \text{GV}\left(I\right)∶=\frac{1}{M}\underset{m}{\sum }I^{2}\left(m\right)-\text{GM}{\left(I\right)}^{2}, \end{array}$$
where *M* is the total number of pixels/voxels and *m* represents the different pixel/voxel positions on the image/volume *I*.
(v) The global covariance of images/volumes *I* and $\tilde{I}$ is defined as
  (B.5) $$\begin{array}{c} \text{GCV}\left(I,\tilde{I}\right)∶=\frac{1}{M}\underset{m}{\sum }I\left(m\right)\tilde{I}\left(m\right)-\text{GM}\left(I\right)\text{GM}\left(\tilde{I}\right), \end{array}$$

where *M* is the total number of pixels/voxels and *m* represents the different pixel/voxel positions on the image/volume *I*.
